# Stretchable Ag electrodes with mechanically tunable optical transmittance on wavy-patterned PDMS substrates

**DOI:** 10.1038/srep46739

**Published:** 2017-04-24

**Authors:** Eun-Hye Ko, Hyo-Joong Kim, Sang-Mok Lee, Tae-Woong Kim, Han-Ki Kim

**Affiliations:** 1 Kyung Hee University, Department of Advanced Materials Engineering for Information and Electronics, 1 Seocheon, Yongin, Gyeonggi-do, 446-701, Republic of Korea; 2 OLED R&D Center, Samsung Display, 230 Nongseo-dong, Yongin-si, Gyeonggi-do, 17113, Republic of Korea

## Abstract

We report on semi-transparent stretchable Ag films coated on a wavy-patterned polydimethylsiloxane (PDMS) substrate for use as stretchable electrodes for stretchable and transparent electronics. To improve the mechanical stretchability of the Ag films, we optimized the wavy-pattern of the PDMS substrate as a function of UV-ozone treatment time and pre-strain of the PDMS substrate. In addition, we investigated the effect of the Ag thickness on the mechanical stretchability of the Ag electrode formed on the wavy-patterned PDMS substrate. The semi-transparent Ag films formed on the wavy-patterned PDMS substrate showed better stretchability (strain 20%) than the Ag films formed on a flat PDMS substrate because the wavy pattern effectively relieved strain. In addition, the optical transmittance of the Ag electrode on the wavy-patterned PDMS substrate was tunable based on the degree of stretching for the PDMS substrate. In particular, it was found that the wavy-patterned PDMS with a smooth buckling was beneficial for a precise patterning of Ag interconnectors. Furthermore, we demonstrated the feasibility of semi-transparent Ag films on wavy-patterned PDMS as stretchable electrodes for the stretchable electronics based on bending tests, hysteresis tests, and dynamic fatigue tests.

Stretchable electronics have attracted great interest for stretchable displays, photovoltaics, touch panels, transistors, sensors, human-health monitoring devices and smart gloves^1^,^2^,^3^,^4^,^5^,^6^,^7^. The fabrication of mechanically stretchable and optically transparent electrodes is very important for obtaining high-performance stretchable electronic devices, because the performance of stretchable electronic devices is critically affected by the mechanical, electrical, and optical properties of the electrodes. In general, transparent conductive materials, including Sn-doped In_2_O_3_, Al-doped ZnO, F-doped SnO_2_, carbon nanotubes (CNT), graphene, Ag nanowires, Cu nanowires, Ag grids, Cu-grids, poly(3,4-ethylene dioxylene thiophene):poly (styrene sulfonic acid) (PEDOT:PSS), and self-assembled Ag networks coated on various substrates have been widely used as transparent electrodes for rigid or flexible optoelectronic devices^8^,^9^,^10^,^11^,^12^,^13^,^14^,^15^,^16^,^17^,^18^. However, typical transparent electrodes coated on glass or PET substrates cannot act as stretchable electrodes for stretchable electronics due to strain limitations. Although graphene, CNT, graphene, and PEDOT electrodes have a fairly high strain to failure, the limited stretchability of the PET substrate made it necessary to develop a high quality stretchable substrate coated with a stretchable electrode. To date, the most commonly used stretchable substrate is polydimethylsiloxane (PDMS), which is prepared by mixing an elastomeric PDMS solution and curing agent at a specific ratio^19^,^20^. A PDMS substrate with surface wrinkles that can relieve mechanical stress has been employed as a stretchable substrate to achieve devices with better stretchability^21^,^22^. Several methods have been reported for making wrinkled or wavy-patterns such as heating, solvent swelling, mechanical stretching/compression, and UV or thermal curing^23^. Yu *et al*., reported that UV treatment on pre-strained PDMS led to the formation of wavy patterns because the surface of the pre-strained PDMS was converted from siloxane to silicon oxide, and cross-linked PDMS was formed though a UVO reaction^24^. Eventually, the imbalance between the surface and bulk on the PDMS resulted in a wrinkle pattern on the surface of the PDMS when the strain was removed from the pre-strained PDMS^25^,^26^. Jun *et al*., reported thick Ag wrinkled electrodes on the oxygen plasma and Sodium Dodecyl treated PDMS substrates as a metal electrode for wearable wireless sensor^27^. Feng *et al*. also reported stretchable CNT transistors on pre-strained PDMS substrates and showed stretchability of the CNT transistor up to 50%^28^. Although wrinkled or wavy-patterning methods for PDMS substrates have been previously reported, there have been no reports on the electrical, optical, and mechanical properties of very thin Ag films coated on wavy-patterned PDMS substrates as stretchable and semi-transparent electrodes for stretchable and transparent interconnectors or stretchable and transparent thin film heaters.

In this work, we reports on the characteristics of thin Ag films coated on wavy-patterned PDMS substrates to use as stretchable electrodes. We compared the mechanical properties of thin Ag electrodes sputtered on flat PDMS and wavy-patterned PDMS substrates, and we suggest that thin Ag films on wavy-patterned PDMS are feasible for stretchable electrodes. The effect of Ag thickness on the flexibility and stretchability of Ag electrodes was investigated in detail using bending tests and dynamic bending fatigue tests. In addition, we suggested that the optical transmittance of the Ag electrode on wavy-patterned PDMS substrate could be tuned by stretching the wavy-patterned PDMS substrate from 0 to 20%. For a precise pattern of the semi-transparent Ag interconnectors, the wavelength and amplitude of the wavy-pattern of PDMS substrate were controlled by the extent of pre-strain and UV-ozone treatment time. Furthermore, we demonstrated the outstanding stretchability of semi-transparent Ag films in two applications: stretchable and semi-transparent interconnectors and semi-transparent electrodes for stretchable and transparent thin film heaters.

## Results

**Figure 1:**
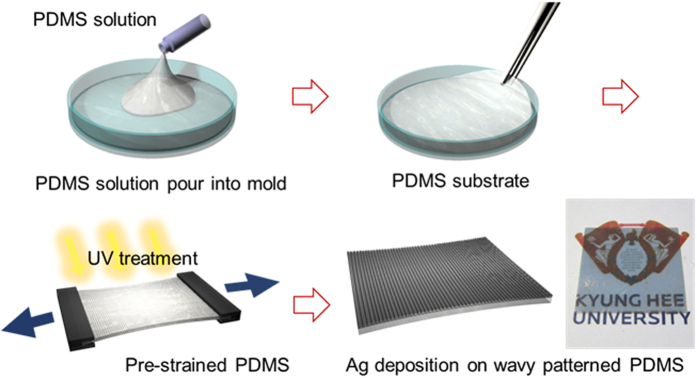
Schematic process to fabricate wavy-patterned stretchable PDMS substrate and semi-transparent Ag films. The photo shows the semi-transparent Ag film coated on a wavy-patterned PDMS substrate.

**Figure 2:**
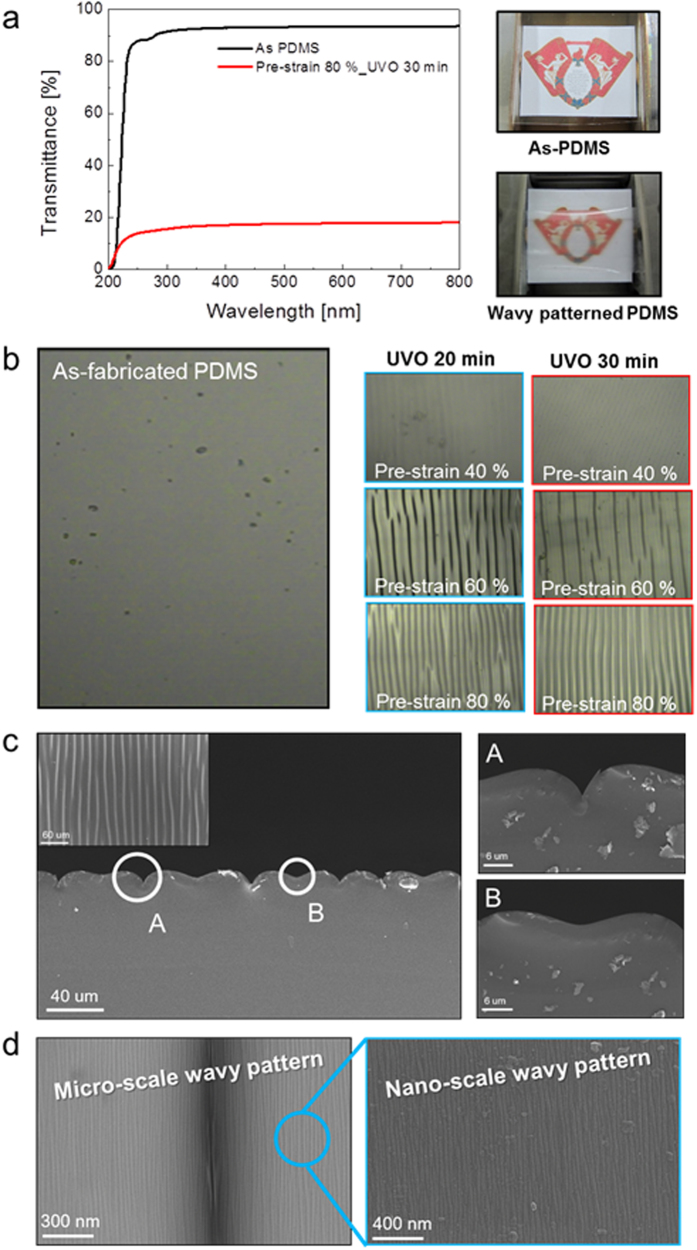
(**a**) Optical transmittance of flat-PDMS and wavy-patterned PDMS substrate. Right side photos exhibit the transparency of the reference flat PDMS and wavy-patterned PDMS substrates. (**b**) Optical microscope images of the flat PDMS and wavy-patterned PDMS substrate fabricated under different UVO irradiation times and pre-strains from 40 to 80%. (**c**) Cross-sectional FESEM images of wavy-patterned PDMS with an inset surface FESEM image. “A” and “B” indicates the sharp hollow and broad hollow buckles on the wavy-patterned PDMS substrate, respectively. (**d**) Enlarged surface FESEM image obtained from the buckle region showing micro-sized and nano-sized buckles.

Figure 1 illustrates the overall process used to fabricate the semi-transparent Ag electrode deposited on a wavy-patterned PDMS substrate. A picture of the stretchable Ag electrode is also provided. We can fabricate semi-transparent stretchable Ag electrodes on a wavy-patterned PDMS substrate by UVO irradiation on a pre-strained PDMS substrate with subsequent sputtering of the Ag film. Figure 2a shows a comparison of optical transmittance of flat PDMS and wavy-patterned PDMS substrates before coating of semi-transparent Ag films. The right side picture shows the transparency of the flat PDMS and wavy-patterned PDMS in the stretching system. The wavy-patterned PDMS sample showed a lower optical transmittance of 17.66% than the flat PDMS substrate (93.39%) due to scattering of light on the wrinkles of the wavy pattern. Figure 2b exhibits an optical microscope (OM) surface image of the flat PDMS and wavy-patterned PDMS prepared at different pre-strain conditions of 40, 60, and 80% under UVO irradiation times of 20 and 30 min. As expected from the optical transmittance measurements, the flat PDMS surface showed a featureless clean surface and a smooth surface morphology. However, the wavy-patterned PDMS substrate showed buckling that occurred perpendicular to the direction of pre-strain. As Yu *et al*. discussed, the extent of buckling on the PDMS substrate depended heavily on the extent of pre-strain and UVO irradiation time^24^. Cross-links on the surface region of the PDMS substrate are significantly affected by UVO irradiation time. Therefore, an increase in UVO irradiating time increased the buckling on the PDMS substrate. The extent of pre-strain of the PDMS influenced the depth of buckling, as shown in Fig. 2b. When the pre-strain applied to the PDMS substrate was removed after UVO treatment, the pre-strained PDMS substrate returned to its equilibrium configuration. However, the difference between the equilibrium strain of the cross-linked top surface region and the soft bottom PDMS substrate resulted in wrinkling at the surface of the PDMS substrate^25^,^26^. The number of hierarchical wrinkles increased by increasing the degree of pre-strain from the initial length of the PDMS substrate. Therefore, we determined that UVO treatment of the PDMS substrate for 30 minutes under a pre-strain of 80% results in an appropriate stretchable substrate for stretchable electrodes. Figure 2c shows a cross-sectional FESEM image of the wavy-patterned PDMS substrate including the buckling system. There are two types of buckles on the surface region of the wavy-patterned PDMS substrate. One is a sharp hollow buckle (A), and the other is a dull hollow buckle (B). The buckling type depends on the pre-strain removal rate of the PDMS substrates. As the strain removal rate decreased, the number of dull, hollow buckles (B) caused by defects increased significantly^29^. In addition, the enlarged surface FESEM image obtained from sharply hollow buckles exhibited numerous nano-sized sub-buckles on the micro-sized buckles (Fig. 2d).

**Figure 3:**
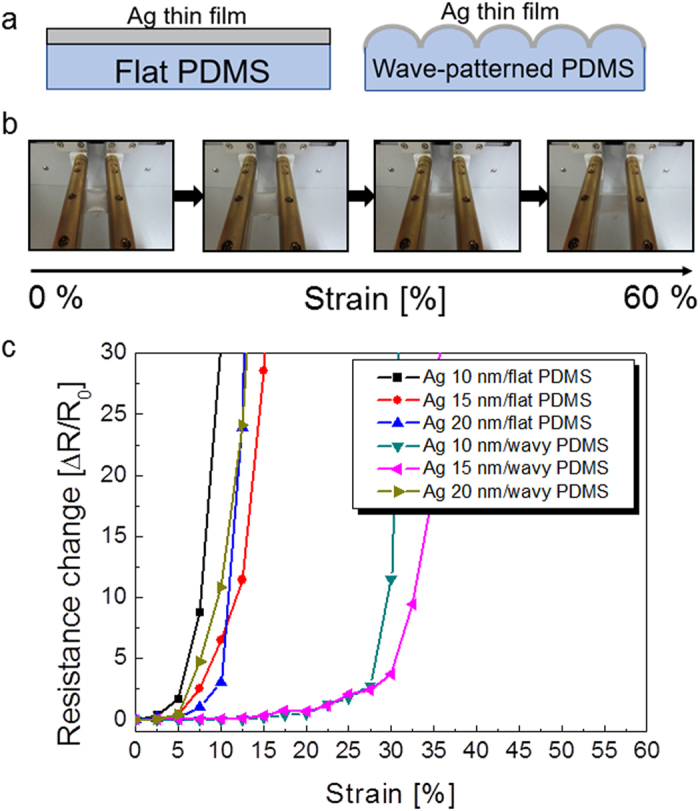
(**a**) Schematic of semi-transparent Ag films on flat PDMS and wavy-patterned PDMS substrates. (**b**) Pictures show the stretching steps for semi-transparent Ag coated PDMS substrates. (**c**) Resistance change of semi-transparent Ag coated on flat PDMS and wavy-patterned substrates as a function of Ag thickness.

**Figure 4:**
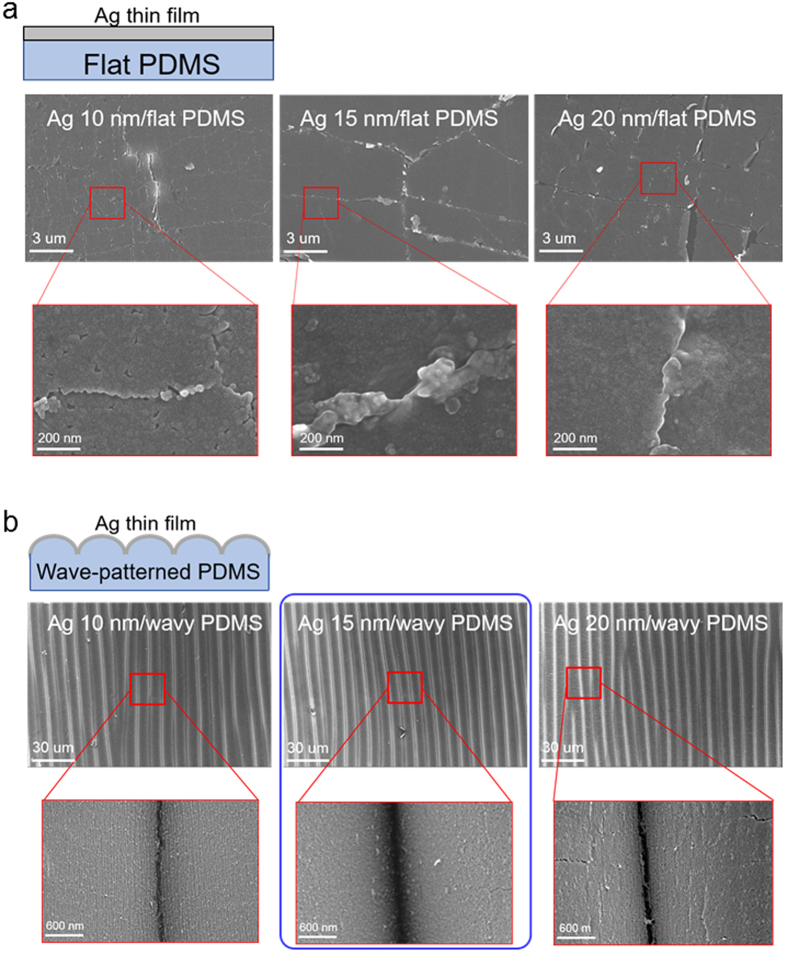
Surface FESEM images of semi-transparent Ag films on (**a**) flat PDMS substrate and (**b**) wavy-patterned PDMS substrates after stretching of the samples up to a strain of 20%.

After fabrication of the wavy-patterned PDMS substrate, a semi-transparent Ag film was coated by using a DC magnetron sputtering system. The upper panel of Fig. 3a shows the schematic structure of semi-transparent Ag films on flat PDMS and wavy-patterned PDMS substrates. The mechanical stretchability of the Ag films coated on the flat and wavy-patterned PDMS substrate was investigated as a function of Ag thickness (10, 15, 20 nm). Figure 3b demonstrates the specially designed stretching test system with a mounted Ag coated wavy-patterned PDMS substrate. The strain was gradually applied to the PDMS substrate from 0% to 60%. Figure 3a shows the stretching test results of the semi-transparent Ag film coated on flat and wavy-patterned PDMS substrates. The change in resistance of the electrode during substrate stretching can be expressed as ΔR(=R − R_0_)/R_0_, where R_0_ is the initial measured resistance, and R is the resistance measured under stretched conditions^30^. In the case of the Ag film on flat PDMS, the whole sample showed an abrupt resistance change within 10% strain due to the formation and propagation of cracks regardless of the Ag thickness. However, the 10 and 15 nm thick Ag films on the wavy patterned PDMS substrate showed a constant resistance change until a strain of 20%. A further increase in strain up to 30% resulted in an increase in resistance change, indicating the formation and propagation of cracks in the Ag. The high strain of the Ag film on the wavy patterned PDMS substrate indicated the possibility of using a semi-transparent Ag film as a stretchable electrode. However, as the thickness of the Ag increased to 20 nm, the semi-transparent Ag film on wavy-patterned PDMS showed similar mechanical properties to the Ag films on flat PDMS substrates. At a strain of 10%, the sample showed an abrupt increase in the change in resistance. The change in surface morphology of the semi-transparent Ag film on flat and wavy-patterned PDMS substrates after stretching was analyzed by FESEM in order to investigate the failure mechanism during the stretching test. Figure 4a shows the surface FESEM image of the semi-transparent Ag film on a flat PDMS substrate as a function of Ag thickness after the 15% stretching test. As expected from the stretching test in Fig. 3a, all Ag films on flat PDMS substrates showed severe cracks which occurred in random directions. Enlarged FESEM images also clearly showed cracks separating the Ag layer. The cracks resulted in disconnection in the conduction paths in the Ag film and increased the resistance change in the thin Ag layer. The surface FESEM images in Fig. 4b show the surface morphology of the Ag film on the wavy-patterned PDMS substrate after stretching to 20% strain. The 10 and 15 nm thick Ag films on wavy-patterned PDMS substrates showed a smooth surface morphology without cracks even though they experienced 20% strain. However, the 20 nm thick Ag film showed some cracks on the broad, hollow buckling region, which separated from the Ag film when the PDMS substrate was stretched. The abrupt resistance change in the 20 nm thick Ag film on the wavy-patterned PDMS substrate was attributed to the formation of cracks in the sub-buckle region.

**Figure 5:**
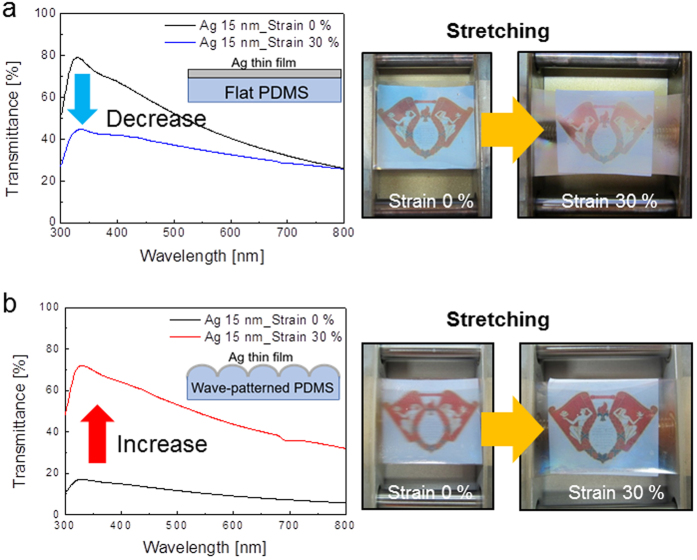
Optical transmittance of (**a**) Ag electrodes on flat PDMS substrate and (**b**) Ag electrodes on wavy PDMS substrate before and after stretching of 30%.

Figure 5 shows the optical transmittance change for the semi-transparent Ag film on flat PDMS and on the wavy-patterned PDMS substrate during the stretching test. The 15 nm thick Ag film on a flat substrate (Fig. 5a) showed a decrease in optical transmittance from 42.59 to 32.98% when the substrate was stretched to 30%. The long-wavelength transmittance of the Ag film on the flat PDMS substrate decreased because the Ag metal absorbs long-wavelength light. The picture on the right side shows a decrease in optical transparency after stretching of the PDMS substrate. However, the Ag film on the wavy-patterned PDMS substrate in Fig. 5b showed an increase in optical transmittance when the substrate was stretched to 30% more than its original length. The transmittance of the Ag electrode deposited on the wavy-patterned PDMS substrate showed a low value of 9.48% in the wavelength range from 400 nm to 800 nm. However, as both ends of the electrode stretched, the optical transmittance of the Ag film on the wavy-patterned PDMS substrate increased and was 45.27% on an average for wavelengths from 400 nm to 800 nm. This change in transmittance occurred because the Ag film on the wavy-patterned PDMS became straighter, which reduced the light scattered due to buckling. The pictures also show a change in the transparency of the Ag film due to substrate stretching. The change in optical transmittance by stretching indicates that the deposition of a semi-transparent Ag film coated on wavy-patterned PDMS resulted in an electrode materials with an optical transparency that can be tuned using an external force. The optical transmittance values of devices have been tuned by an applied electric field in electrochromic devices or by an applied voltage in polymer dispersed liquid crystals (PDLCs)^31^,^32^. However, the optical transparency of our Ag film on a wavy-patterned PDMS substrate was tunable by means of an applied external force. Detailed optical and electrical properties of Ag films on flat PDMS and wavy-patterned PDMS substrates before and after stretching (30%) are summarized in Table 1. Both samples showed a fairly low sheet resistance before stretching of the PDMS substrates. The sheet resistance of the semi-transparent Ag film on a flat PDMS substrate significantly increased after stretching of the substrate to about 30% strain. The stretched Ag electrode showed a high sheet resistance due to the formation and propagation of cracks as well as thinning of the A layer that occurred during stretching. However, the Ag electrode on the wavy-patterned PDMS substrate showed a fairly low change in sheet resistance after 30% stretching because the external force was relaxed as the wave pattern unfolded. Therefore, the Ag film on the wavy-patterned PDMS substrate retained its low sheet resistance and showed increased optical transmittance when it was stretched up to 30%.

**Figure 6:**
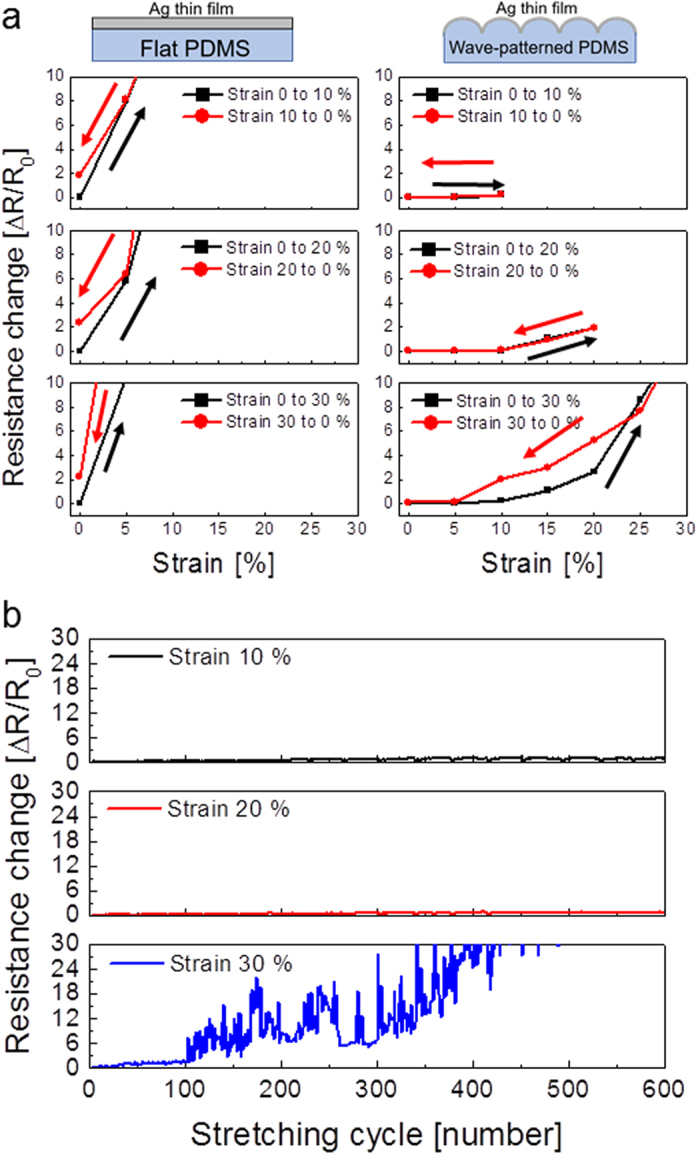
(**a**) Hysteresis of resistance change during stretching test of the Ag films on flat and wavy patterned PDMS substrates when samples were stretched from 0 to 30%. (**b**) Dynamic fatigue stretching test of the Ag films on a wavy-patterned PDMS substrate as a function of stretching cycles.

Figure 6a shows the hysteresis of resistance change for the samples as the substrates were stretched. To compare the recovery properties of the stretched Ag electrodes on the PDMS substrates, we measured the resistance change of the Ag film before and after stretching from 10 to 30%. All samples of the Ag film on a flat PDMS substrate showed significantly increased resistance after stretching due to cracking in the Ag film. However, the resistance change in the Ag film on the flat PDMS substrate recovered somewhat after the strain was removed because physical contact was restored between cracks in the Ag film. Once the cracks formed on the Ag film during stretching, there was permanent increase in the resistance of the Ag film even after the cracked Ag film returned to its original position. In contrast, the resistance of the Ag electrode on the wavy patterned PDMS substrate is fairly constant at 10 and 20% strain due to effective relaxation of the strain via the wavy pattern. When the strain was removed, the resistance of the Ag electrode returned to its initial resistance value. At 30% strain, this Ag electrode showed an increased resistance due to the formation cracks on the sub-wavy pattern region as expected from Fig. 4. Therefore, when the strain was removed, the Ag electrode showed higher resistance than the resistance measured as strain increased. Figure 6b shows the dynamic fatigue stretching test results of the 15 nm thick Ag electrode on the wavy-patterned PDMS substrate as a function of strain from 10 to 30%. The Ag electrode that was dynamically stretched at 10 and 20% showed a constant resistance change with increasing stretching cycles, demonstrating the superior stretchability of the Ag electrode on the wavy-patterned PDMS substrate. However, dynamic fatigue stretching test results at 30% strain showed a large change in resistance after 600 stretching cycles due to severe crack formation and separation of cracked Ag films. We found that the semi-transparent Ag film on the wavy-patterned PDMS substrate endured the stretching up to 20% without a change in resistance based on the dynamic fatigue test results.

**Figure 7:**
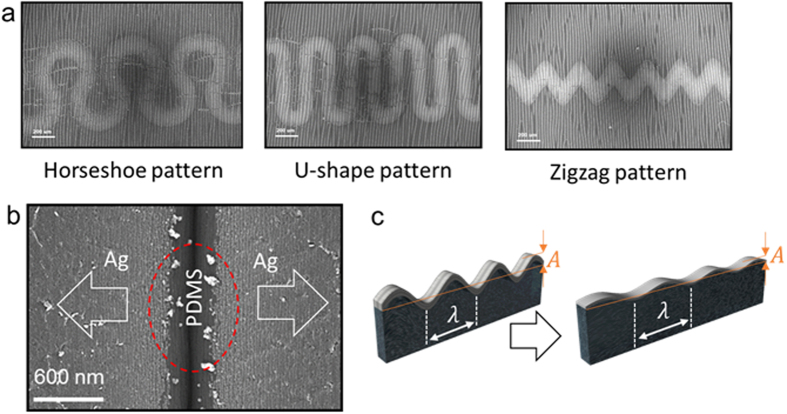
Surface FESEM images of horseshoe, U-shape, and zigzag patterns fabricated on the wavy-patterned PDMS substrates with a deep buckling. (**b**) Surface FESEM image of the horseshoe patterned Ag interconnector after stretching 20%. The dotted circle line indicates the uncoated region in the deep buckling region of the PDMS substrate. (**c**) Decrease in amplitude of a wavy-pattern on the surface region of PDMS substrate by decreasing pre-strain.

Although the semi-transparent Ag film fully covered the wavy-patterned PDMS substrate showed a constant resistance until a strain of 20%, the horseshoe, U-shape, and zigzag patterned Ag electrodes by shadow metal mask in Fig. 7a demonstrated very high sheet resistance or insulting properties. The insulating property of the horseshoe shape, U-shape, and zigzag-patterned Ag electrodes on the wavy patterned PDMS substrate could be attributed to the uncoated area in a sharp hollow buckling region as circled in Fig. 7b. Because the uncoated region completely separated the wrinkled Ag electrode during stretching, the patterned Ag electrode with specific interconnector shapes behave like an insulator. Therefore, to improve the conductivity of wrinkled semi-transparent Ag electrode, the wavelength and amplitude of the wavy pattern should be modified like Fig. 7c by increasing UV/ozone treatment times.

**Figure 8:**
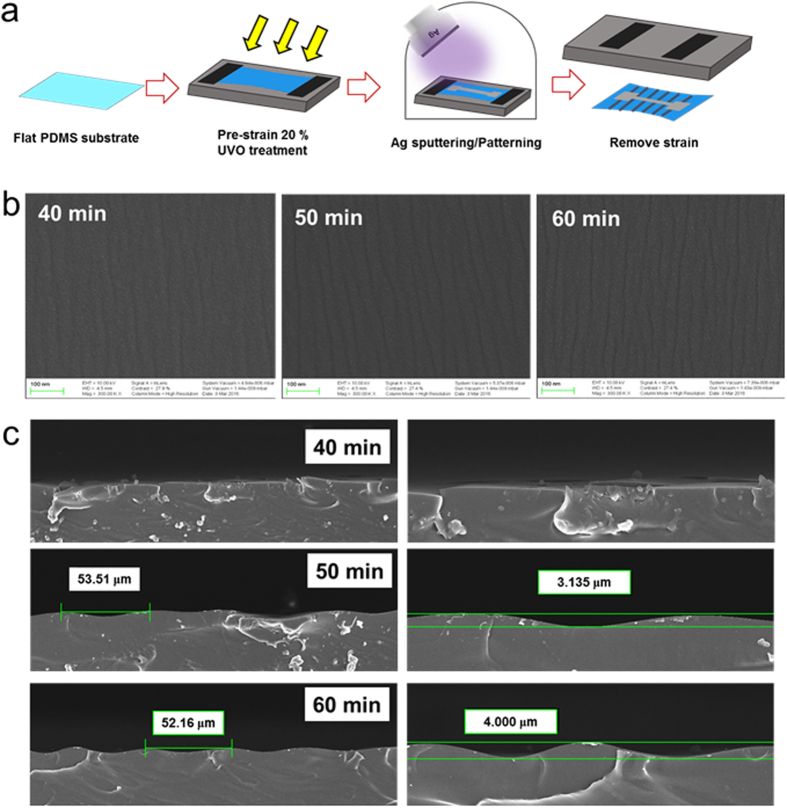
(**a**) Schematic process to fabricate semi-transparent Ag interconnectors (horseshoe, U-shape, and zigzag patterns) on wavy-patterned PDMS substrate with a smooth buckling. (**b**) Surface and (**c**) cross-sectional FESEM images of the wavy-patterned PDMS substrates fabricated at a constant pre-strain of 20% as a function of UV/ozone treatment time (40, 50, 60 min).

**Figure 9:**
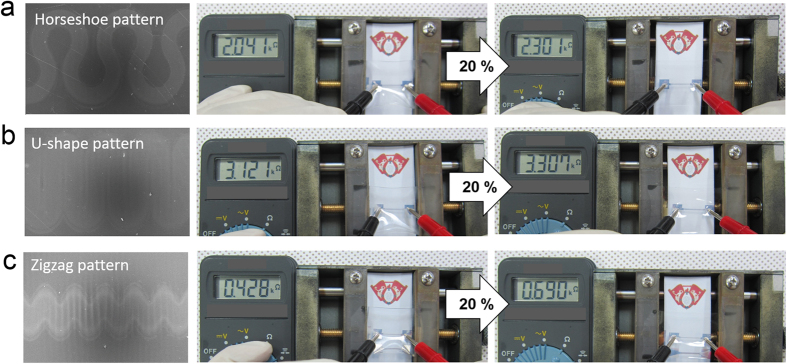
Surface FESEM images and stretchabillity of (**a**) horseshoe, (**b**) U-shape, and (**c**) zigzag patterned Ag interconnectors fabricated on the wavy-patterned PDMS substrates with smooth buckling.

To fabricate a wavy-pattern with a smooth buckling, we prepared PDMS substrates under reduced pre-strain of 20% as a function of UV/ozone treatment time from 40 to 60 min. Figure 8a showed schematic fabrication processes to prepare patterned Ag interconnectors on the wavy-patterned PDMS substrate with a smooth buckling. To reduce hierarchical wrinkles, we decreased the extent of pre-strain (20%) and increased the UV/ozone treatment time. Figure 8b shows a surface FESEM images of the wavy-patterned PDMS prepared under reduced pre-strain of 20% with increasing UV/ozone treatment time. As we expected from Fig. 2b, the depth of bulking on PDMS substrate was significantly reduced even after the pre-strain applied to the PDMS substrate was removed. Figure 8c also demonstrate cross-sectional FESEM images of the wavy-patterned PDMS substrate with increasing UV/ozone treatment time. Because the depth of buckling was mainly affected by the extent of pre-strain, all samples prepared at a constant pre-strain of 20% showed much smoother buckling than the PDMS prepared under pre-strain of 60 or 80% (Fig. 2c). In addition, an increase in UV/ozone treatment time resulted in an increase in amplitude of the buckling due to increased cross-links on the surface region. Figure 9a–c shows the surface FESEM images on wavy-patterned PDMS substrate with a smooth buckling and resistance change of the horseshoe, U-shape, and zigzag-patterned Ag interconnectors before and after stretching (20%). Unlike insulating properties of the patterned Ag interconnectors on wavy-patterned PDMS substrates with a deep buckling, the measured resistance from the horseshoe, U-shape, and zigzag-patterned Ag interconnectors on the wavy-patterned PDMS with a smooth buckling indicated good connectivity of the patterned Ag interconnectors. Because the depth of buckling was significantly reduced, all patterned Ag interconnectors showed a small change in measured resistance even after stretching of 20% regardless of the pattern shapes. Therefore, we concluded that the wavy-patterned PDMS substrate with a smooth buckling is favorable to stretchable and semi-transparent Ag interconnectors with specific stretchable patterns.

**Figure 10:**
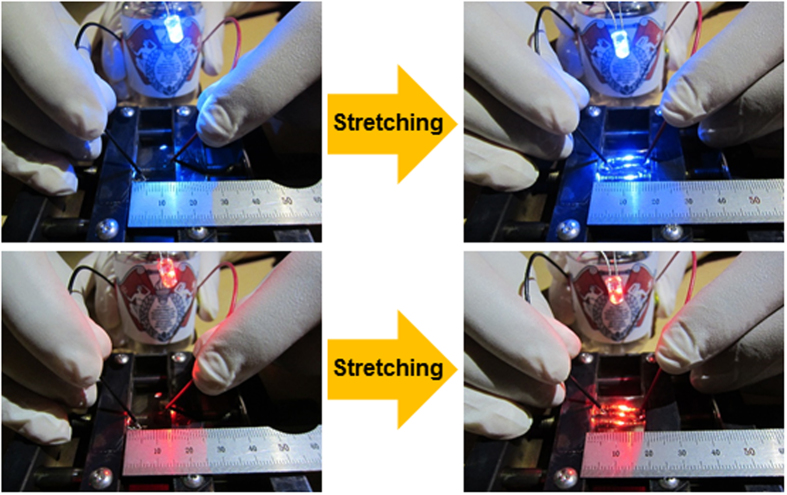
Applications of the semi-transparent Ag electrodes on wavy patterned PDMS substrates. Stretchable interconnector connecting blue and red LEDs with increasing Ag electrode length up to a strain of 20%.

**Figure 11:**
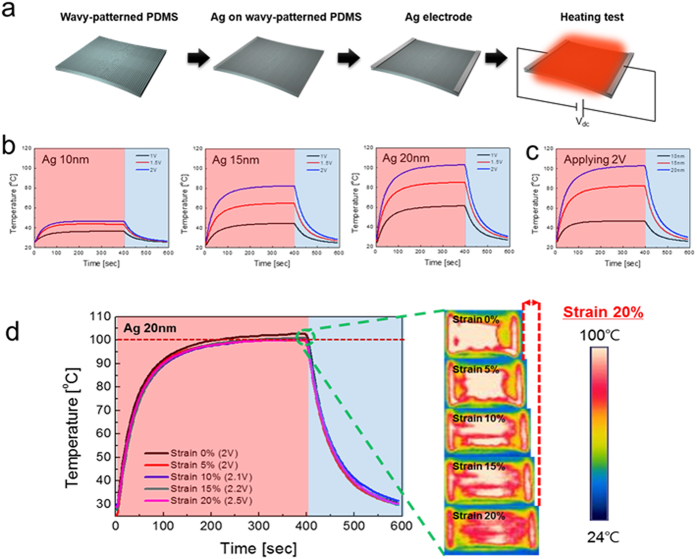
(**a**) Schematic of the fabrication process of stretchable tin film heaters. (**b**) Temperature profile of stretchable TFHs as a function of semi-transparent Ag electrode thickness under operation at different input voltages. (**c**) Temperature profile of stretchable TFHs at a 2 V input voltages with increasing thickness of semi-transparent Ag electrode. (**d**) Temperature profile of stretchable thin film heaters as a function of strain up to 20% while operating. The picture on the right shows an IR image of the stretched thin film heater.

Figure 10 shows the feasibility of our semi-transparent Ag films on wavy-patterned PDMS substrate as stretchable interconnectors. The Ag electrode was connected to conventional blue and red light emitting diodes and was slowly stretched until the LEDs turned off. The LEDs were stable until the Ag electrode fabricated on wavy-patterned PDMS substrate was stretched up to 20%, indicating that current flowed through the stretched Ag electrode up to this point. Stretchable Ag electrodes fabricated on PDMS substrate with a smooth buckling can also be used as stretchable TFHs as shown in Fig. 11. As shown in the upper panels of Fig. 11a, semi-transparent and stretchable TFHs were fabricated on the semi-transparent Ag film with sizes of 25 × 25 mm^2^ using a two-metal terminal side Ag contact configuration. Figure 11b shows the temperature profiles of the stretchable TFHs with a semi-transparent stretchable Ag electrode as a function of thickness. In general, as the input voltage was increased, the temperature of the TFHs increased, as shown in the equation below^33^.


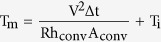


Here, h_conv_ is a convective heat transfer coefficient, A_conv_ is the surface area, and T_m_ and T_i_ are the maximum and initial temperature, respectively. Based on the equation above, it is apparent that the maximum temperature of the TFHs decreases with increasing resistance (R) under a constant input DC voltage (V). An increase in thickness of semi-transparent Ag electrode led to a higher saturation temperature as shown in Fig. 11b, due to lower resistance of the semi-transparent Ag electrode. The TFHs fabricated on semi-transparent Ag electrode with a thickness of 20 nm reached 100 °C when the DC voltage supplied was 2 V. The TFHs with thicker Ag electrode showed a higher saturation temperature at a same input voltage (2 V) as shown in Fig. 11c. The temperature of stretchable TFHs with a 20 nm thick Ag electrode was plotted as a function of strain from 0 to 20% at input voltage range of 2.0–2.5 V. In the case of the stretchable TFHs on a wavy patterned PDMS substrate without strain (0%), the temperature of TFH reached 100 °C at a constant voltage of 2 V due to low resistance of semi-transparent Ag electrode. Even after increasing strain from 0 to 20%, the maximum temperature of stretchable TFHs was kept at 100 °C. A slight increase in the resistance by stretching led to an increase in the input voltage from 2 V to 2.5 V because the maximum temperature of the TFHs is closely related to the electrode resistance as shown in the equation above. However, even at a high strain of 20%, we observed successful operation of the TFHs which indicates that semi-transparent and stretchable Ag film on a wavy-patterned PDMS substrate can be used as high performance stretchable electrodes.

## Conclusions

In summary, we developed semi-transparent and stretchable Ag films on wavy patterned PDMS substrates as stretchable electrodes. Using UVO irradiation on a pre-strained PDMS substrate and subsequent sputtering of the Ag film, we fabricated stretchable Ag electrodes for stretchable interconnects and stretchable TFHs. A semi-transparent Ag films was formed on the wavy-patterned PDMS substrate and this showed better stretchability (strain 20%) than the Ag film formed on the flat PDMS substrate due to the effective release the strain relaxation in the wavy-pattern. In addition, the optical transmittance of the Ag electrode on the wavy-patterned PDMS substrate was tunable based on the degree of stretching in the PDMS substrate. It was found that wavy-patterned PDMS substrate with a smooth buckling was beneficial for precisely patterned Ag interconnectors because the depth of buckling critically affected on the connectivity of stretchable Ag interconnectors. Furthermore, several bending tests, hysteresis tests, and dynamic fatigue tests were used to demonstrate the feasibility of using semi-transparent Ag films on wavy-patterned PDMS as a stretchable electrode for stretchable interconnectors and stretchable TFHs.

## Methods

### Fabrication of wavy-patterned PDMS substrate

Transparent and stretchable PDMS substrates were prepared by mixing elastomeric PDMS and curing agent at a ratio of 10:1 by mass. After degassing, the mixed solution was poured into a circular-shaped mold to make a stretchable substrate. The mixed solution in the mold was cured at a constant temperature of 60 °C for more than an hour to form a PDMS substrate with a thickness of ~300 μm. The cured PDMS substrate was ripped off from the circular-shaped mold, and then the PDMS substrate was cut into rectangular shapes with sizes of 4 × 2.5 cm^2^. We designed a special PDMS substrate holder to hold the stretched-PDMS substrate at a constant length. This holder was used in the UV ozone (UVO) treatment system or loaded into the sputtering chamber. To fabricate a wavy-patterned PDMS substrate, the end of the rectangular PDMS substrate was fastened on the substrate holder (Figure S1, Supporting Information) and both ends of the PDMS substrate were pulled until the desired pre-strain condition was reached. The surface of the pre-strained PDMS substrate was then exposed to UVO for 30 min. After UVO treatment, the pre-strained PDMS substrate was released at a rate of 1 mm/sec to form a wavy pattern on the PDMS substrate (Fig. 1). To fabricate wavy-patterned PDMS substrate with a smooth buckling, the PDMS substrate was fastened on substrate holder at a constant pre-strain of 20% with increasing UV/ozone treatment time from 40 to 60 min.

### DC sputtering of semi-transparent Ag film on the wavy patterned PDMS

Semi-transparent Ag films were deposited on the wavy-patterned and flat PDMS substrates at various thickness (10, 15, 20 nm) using DC magnetron sputtering system. Within the optical skin depth (12 nm), the Ag thin film showed fairly high optical transmittance even though bulk Ag is opaque. During the DC sputtering process, the PDMS substrates were constantly rotated at a speed of 20 rpm at room temperature. The semi-transparent Ag films were grown at a constant DC power of 100 W, a working pressure of 2 m Torr, and an Ar flow rate of 20 sccm.

### Characterization of the stretchable Ag electrodes deposited on the PDMS substrate

The electrical and optical properties of the Ag films on the wavy-patterned PDMS substrate were examined using a four point probe and a UV/visible spectrometer (UV 540, Unicam). These data were compared with the Ag film coated on a flat PDMS substrate before and after stretching. The surface morphology of the Ag films on the wavy-patterned PDMS substrate were examined using field effect scanning electron microscopy (FESEM, LEO SUPRA 55). The mechanical properties including stretchability, hysteresis, and dynamic fatigue of semi-transparent Ag films on wavy-patterned and flat PDMS substrates were investigated using a custom bending and stretching system. The hysteresis of the stretched Ag films on the wavy-patterned PDMS substrate were measured in the strain range from 0 to 30%. In addition, dynamic fatigue stretching tests were performed using a lab-designed cyclic stretching test machine operated at a frequency of 0.5 Hz for 10,000 cycles.

### Fabrication and evaluations of the stretchable thin film heaters

To demonstrate the feasibility of using the semi-transparent Ag films on a wavy-patterned PDMS substrate as a stretchable electrode for stretchable heaters, conventional TFHs with two-terminal side contacts were fabricated. A 200 nm-thick Ag side contact electrode was sputtered onto the semi-transparent Ag films as a contact electrode. A DC voltage was supplied by a power supply (OPS 3010, ODA technologies) to the semi-transparent Ag films on the wavy-patterned PDMS substrate through a Ag contact electrode at the film edge. The temperature of stretchable TFHs was measured using a thermocouple mounted on the surfaces of the TFHs and an IR thermal imager (A35sc, FLIR).

## Additional Information

**How to cite this article**: Ko, E.-H. *et al*. Stretchable Ag electrodes with mechanically tunable optical transmittance on wavy-patterned PDMS substrates. *Sci. Rep.* **7**, 46739; doi: 10.1038/srep46739 (2017).

**Publisher's note:** Springer Nature remains neutral with regard to jurisdictional claims in published maps and institutional affiliations.

## Supplementary Material

Supporting Information

## Figures and Tables

**Table 1:** Optical and electrical properties of Ag electrodes on the flat PDMS and wavy-patterned PDMS substrate before and after stretching of 30%.

	Ag 15 nm/flat PDMS		Ag 15 nm/wavy PDMS	
	As	Stretching	As	Stretching
Transmittance at 550 nm [%]	45.71	34.68	10.22	48.08
Transmittance at 400–800 nm [%]	42.58	32.98	9.48	45.27
Sheet resistance [Ohm/sq.]	9	3600	12	69
