# Supplementary material for: Stretchable Ag electrodes with mechanically tunable optical transmittance on wavy-patterned PDMS substrates
Source: Sci Rep. 2017 Apr 24;7:46739. doi: 10.1038/srep46739 (PMC5402286; doi:10.1038/srep46739)
Supplement: Supporting Information [file srep46739-s1.pdf]

Supporting information

Stretchable Ag electrodes with mechanically tunable optical transmittance on wavy-patterned PDMS substrates

Eun-Hye Ko[a], Hyo-Joong Kim[a], Sang-Mok Lee[a], Tae-Woong Kim[b], and Han-Ki Kim*[,a]

[a]Kyung Hee University, Department of Advanced Materials Engineering for Information and Electronics, 1 Seocheon, Yongin, Gyeonggi-do 446-701, Republic of Korea

[b]OLED R&D Center, Samsung Display, 230 Nongseo-dong, Yongin-si, Gyeonggi-do, 17113, Republic of Korea

*imdlhkkim@khu.ac.kr

Fabrication of wavy-patterned PDMS substrate: Transparent and stretchable PDMS substrate was prepared by mixing of elastomeric PDMS and curing agent at a ratio of 10:1 by mass. **Figure 1S** are pictures demonstrating fabrication process of a wavy-patterned PDMS substrate.

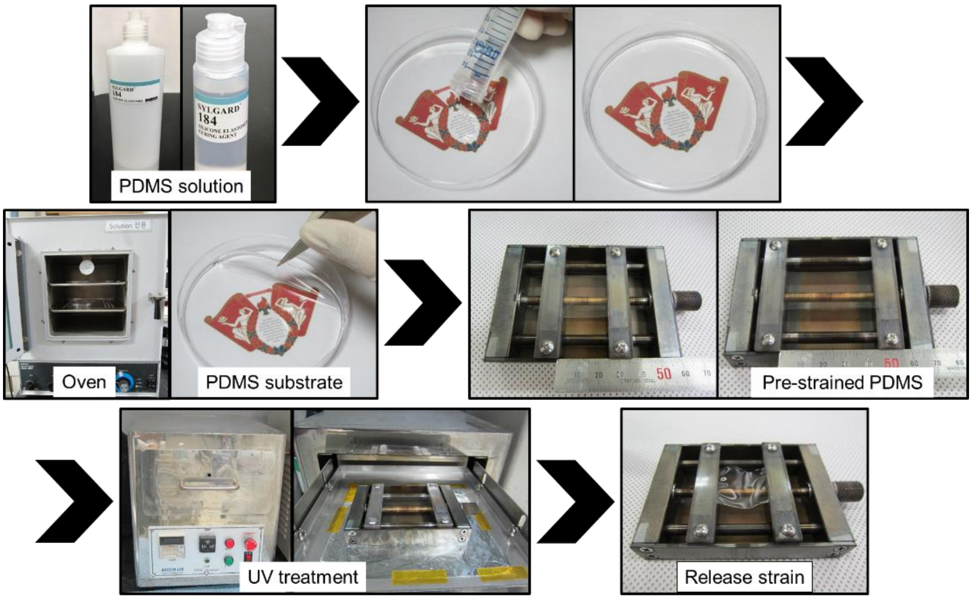

Figure 1S. Fabrication process of a wavy-patterned PDMS substrate by using UVO irradiation on the pre-strained PDMS substrate.

After degassing, the mixed solution was poured into a circular-shaped mold (4 inch) to make stretchable PDMS substrate. The mixed solution in mold was cured in a conventional oven at a constant temperature of 60 °C for more than an hour to form PDMS substrate with a thickness of ~300 μm. The cured PDMS substrate was ripped off from the mold, and then the PDMS substrate was cut into rectangular shapes with sizes of 4×2.5 cm^2. To hold the stretched-PDMS substrate at a constant length, we specially designed PDMS substrate holder as shown in **Figure 1S**. To fabricate wavy-patterned PDMS substrate, the end of the rectangular PDMS substrate was fastened on the substrate holder and pulled both ends of the PDMS substrate up to desired pre-strain condition. The surface of pre-strained PDMS substrate was then exposed to UVO for 30 min in a typical UVO treatment system. After UVO treatment, the pre-strained PDMS substrate was released at a rate of 1 mm/sec to form a wavy pattern on the PDMS substrate.

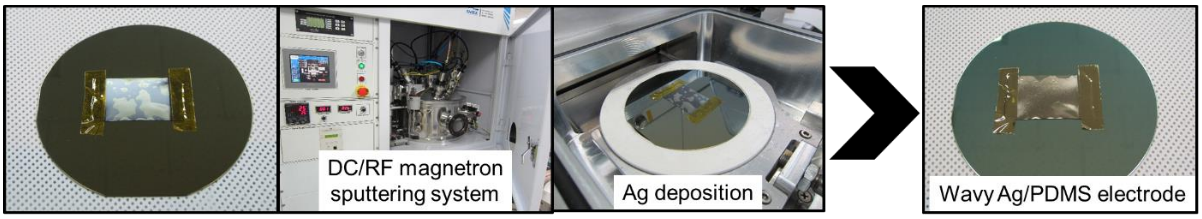

Figure 2S. Sputtering of a semi-transparent Ag film on a wavy patterned PDMS substrate by means of DC magnetron sputtering.

DC sputtering of semi-transparent Ag film on the wavy patterned PDMS: Using DC magnetron sputtering system (Sntek DC magnetron sputtering), a semi-transparent Ag film was deposited on the wavy-patterned and flat PDMS substrates as a function of film thickness (10, 15, 20 nm). Prior to loading of the sample, the wavy-patterned PDMS substrate was tightly attached on the Si wafer as shown in **Figure 2S**. Then the PDMS/Si wafer was load into the

load-lock chamber of the DC sputtering system. During DC sputtering process, the PDMS substrates were constantly rotated at a speed of 20 rpm at room temperature. The semi-transparent Ag films were grown at a constant DC power of 100W, working pressure of 2m Torr, and Ar flow rate of 20 sccm.
